# The number of valvular insufficiency is a strong predictor of cardiovascular and all-cause mortality in hemodialysis patients

**DOI:** 10.1007/s11255-023-03576-3

**Published:** 2023-04-03

**Authors:** Honglan Wei, Shufang Liu, Ming Tian, Weifeng Shang, Hua Li, Yang Wu, Junwu Dong

**Affiliations:** 1 Department of Nephrology, Wuhan Fourth Hospital, Puai Hospital, Tongji Medical College, Huazhong University of Science and Technology, Wuhan, 430030 Hubei China; 2 Department of Ophthalmology, Tongji Medical College, Wuhan Children’s Hospital (Wuhan Maternal and Child Healthcare Hospital), Huazhong University of Science and Technology, Wuhan, Hubei China

**Keywords:** Valvular insufficiency, Cardiovascular mortality, All-cause mortality, Hemodialysis

## Abstract

**Objectives:**

To investigate the relationship between the number of valvular insufficiency (VI) and emergency hospitalization or mortality in maintenance hemodialysis (HD) patients.

**Methods:**

The maintenance HD patients with cardiac ultrasonography were included. According to the number of VI ≥ 2 or not, the patients were divided into two groups. The difference of emergency hospitalized for acute heart failure, arrhythmia, acute coronary syndrome (ACS) or stroke, cardiovascular mortality, and all-cause mortality between the two groups were compared.

**Results:**

Among 217 maintenance HD patients, 81.57% had VI. 121 (55.76%) patients had two or more VI, and 96 (44.24%) with one VI or not. The study subjects were followed up for a median of 47 (3–107) months. At the end of the follow up, 95 patients died (43.78%), of whom 47 (21.66%) patients died because of cardiovascular disease. Age (HR 1.033, 95% CI 1.007–1.061, *P* = 0.013), number of VI ≥ 2 (HR 2.035, 95% CI 1.083–3.821, *P* = 0.027) and albumin (HR 0.935, 95% CI 0.881–0.992, *P* = 0.027) were independent risk factors for cardiovascular mortality. The three parameters were also independent risk factors for all-cause mortality. The patients with number of VI ≥ 2 were more likely to be emergency hospitalized for acute heart failure (56 [46.28%] vs 11 [11.46%], *P* = 0.001). On the contrary, the number of VI was not associated with emergency hospitalized for arrhythmia, ACS or stroke. Survival analysis results showed that probability of survival was statistically different in the two groups (*P* < *0.05*), no matter based on cardiovascular mortality or all-cause mortality. Based on age, number of VI ≥ 2 and albumin, nomogram models for 5-year cardiovascular and all-cause mortality were built.

**Conclusions:**

In maintenance HD patients, the prevalence of VI is prominently high. The number of VI ≥ 2 is associated with emergency hospitalized for acute heart failure, cardiovascular and all-cause mortality. Combining age, number of VI ≥ 2, and albumin can predict cardiovascular and all-cause mortality.

## Introduction

Chronic kidney disease (CKD) is a growing disease burden globally, which is predicted to become the fifth global cause of death by 2040 [1]. In 2017, there were 132·3 million [95% UI 121·8 to 143·7] cases of CKD in China, counting for 18.97% of global [2]. It means that China has become the country with the heaviest burden of CKD in the world. Among them, dialysis expenses account for the main CKD medical expenses. Hemodialysis (HD) is the most common form of renal replacement therapy in the world, accounting for approximately 89% of all dialysis and 69% of all renal replacement therapy [3]. According to the data from Chinese national renal data system (CNRDS), by December 31, 2020, there were 806,759 dialysis patients nationwide, including 692,736 HD patients, which accounting for 85.87%.

Cardiovascular disease (CVD) is one of the leading cause of death among maintenance HD patients [4], which is found in more than half of the cases [5]. Common cardiovascular diseases include coronary artery disease (CAD), arrhythmias, and sudden cardiac death (SCD). Except that, valvular heart disease (VHD) is also the common concomitant disease in dialysis [6]. Rheumatic heart disease and degenerative with age are the main cause of VHD [7]. Over the past 50 years, with the wide use of antibiotics and the development of medicine, the epidemiology of VHD has changed markedly in worldwide. Rheumatic heart disease has significantly decreased. On the contrary, as the population is aging, degenerative valve disease has been gradually increasing [8]. Except that, CKD is the independent risk factor of VHD [9].

Both rheumatic heart disease and degenerative changes may cause valvular insufficiency (VI). With the decline of renal function, the prevalence of VI is increasing, prominently in mitral valve [5]. Except that, valve calcification (VC) is common in CKD. It was reported that 80–99% CKD stage 5 patients had VC [10]. The prevalence of VC is eight times higher in maintenance HD patients than in the general population [11]. While several medical literature have reported that VC was associated with the mortality of maintenance HD patients [12, 13], less attention has been paid to VI. Now, in this study, we aimed to investigate the relationship between the number of VI and the mortality in maintenance HD patients.

## Materials and methods

### Patient selection

The new entry maintenance HD patients in the Blood Purification Department of Wuhan Fourth Hospital from April 1, 2012, to July 15, 2019, were selected. The inclusion criteria: (1) age ≥ 18 years; (2) cardiac ultrasonography was carried out within a week; (3) patients accepted standardized quality control management and regular hemodialysis until December 31, 2020 or died. The primary outcome measures were emergency hospitalized for acute heart failure, arrhythmia, stroke or new onset acute coronary syndrome (ACS), containing angina pectoris or acute myocardial infarction (AMI). The secondary outcome measures were cardiovascular or all-cause mortality. The exclusion criteria: (1) patients had a history of malignancy; (2) patients leave the dialysis center midway because of changing to peritoneal dialysis or kidney transplant; (3) patients lack of complete medical records, and (4) patients had a history of valve intervention or congenital heart disease.

### Data collection

The basic data of patients were collected, including age, gender, using angiotensin converting enzyme inhibitors (ACEI) or angiotensin receptor blocker (ARB), the history of diabetes mellitus (DM), hypertension and CVD, containing acute myocardial infarction and angina. After obtaining the consent from the patient, blood specimens for biochemical tests were collected from the vascular access before dialysis. The baseline clinical data included hemoglobin, serum albumin (ALB), calcium, phosphorus, parathyroid hormone (PTH) and serum uric acid.

Cardiac ultrasonography is a specific and sensitive method for the detection of VI. All cardiac ultrasonography measurements were performed by two sonographers unaware of biochemical results. VI was defined according to guideline recommendations [14].

### Statistics

Data analysis was performed with Statistical Package for Social Analysis (SPSS for Windows, IBM Corp, USA) version 20.0. The study population characteristics were presented as the mean ± standard deviation (SD), or percentage. According to the number of VI ≥ 2 or not, the studies were categorized into two groups. Two-sample *t*-test was used to compare the differences between the two groups for continuous variables. Differences for the nominal variables were compared by Chi-square test. To estimate survival probabilities, the Kaplan–Meier method was used and log-rank test for comparing the differences between the two groups. Cox regression analysis was used to explore the risk factors for new onset ACS, cardiovascular and all-cause mortality. Multivariate logistic regression models were used to test the associations of variables and number of VI ≥ 2. Statistical significance was defined as *P* < 0.05. GraphPad Prism 8 was used in graphic production. Nomogram models were performed with R version 4.1.3.

## Results

### Baseline data comparison

A total of 262 patients were selected, in them 15 cases with malignancy, 16 cases changed to peritoneal dialysis, 7 cases received a kidney transplant, 7 cases without complete medical records, were excluded. At last, 217 patients were enrolled in this study. 133 (61.29%) males and 84 (38.71%) females were contained. The mean age was 59.64 ± 14.52 years. There were 177 (81.57%) patients with VI, and 40 (18.43%) patients without. The distribution of VI in the patients are shown in Table 1. There were 121 (55.76%) patients with two or more VI. Based on the number of VI ≥ 2 or not, the studies were divided into two groups. The clinical characteristics of the study patients are shown in Table 1.

**Table 1:** Characteristics of the study population

	Overall population	Number of valve insufficiency		*p* trend
	*n* = 217	< 2 (*n* = 96)	≥ 2 (*n* = 121)	
Age, years	59.64 ± 14.52	58.83 ± 14.71	60.27 ± 14.40	0.470
Man, *n* (%)	133 (61.29)	61 (63.54)	72 (59.5)	0.577
History of DM, *n* (%)	108 (49.77)	52 (54.20)	56 (46.28)	0.276
History of CVD, *n* (%)	63 (29.03)	24 (25.00)	39 (32.23)	0.292
History of hypertension, *n* (%)	207 (95.39)	93 (96.88)	114 (94.21)	0.518
ACEI/ARB use, *n* (%)	157 (72.35)	75 (78.13)	82 (67.77)	0.096
Mitral insufficiency, *n* (%)	141 (64.98)	31 (32.29)	110 (90.90)	0.001
Tricuspid insufficiency, *n* (%)	116 (53.46)	14 (14.58)	102 (84.30)	0.001
Aortic insufficiency, *n* (%)	84 (38.71)	14 (14.58)	70 (57.85)	0.001
Pulmonary valve insufficiency, *n* (%)	37 (17.05)	2 (2.08)	35 (28.93)	0.001
ALB (g/L)	36.49 ± 5.65	37.14 ± 6.00	36.00 ± 5.34	0.150
Hemoglobin (Hb, g/L)	95.38 ± 22.40	100.9 ± 20.61	91.14 ± 22.87	0.001
Ca (mmol/L)	2.18 ± 0.26	2.21 ± 0.24	2.15 ± 0.27	0.060
P (mmol/L)	1.59 ± 0.55	1.60 ± 0.53	1.58 ± 0.57	0.800
PTH (pg/mL)	346.01 ± 357.55	349.41 ± 377.30	346.36 ± 342.97	0.910
Uric acid (mmol/L)	374.73 ± 137.71	379.97 ± 127.12	370.71 ± 145.72	0.620

*DM* diabetes mellitus, *CVD* cardiovascular disease, *ACEI* angiotensin converting enzyme inhibitors, *ARB* angiotensin receptor blocker, *VI* valvular insufficiency, *ALB* serum albumin, *Ca* calcium, *P* phosphorus, *PTH* parathyroid hormone

### Emergency hospitalization and mortality

By December 31, 2020, the study subjects were followed up for a median of 47 (3–107) months. During the follow up, the patients of two groups emergency hospitalized for acute heart failure, arrhythmia, acute coronary syndrome (ACS) or stroke were shown in Table 2. In general, emergency hospital admission rate of VI ≥ 2 group was higher (90.91% vs 59.38%, *P* = 0.001). This difference was mainly due to heart failure hospitalization (Table 2).

**Table 2:** Emergency hospitalization of two groups

	Number of valve insufficiency		*p* trend
	< 2 (*n* = 96)	≥ 2 (*n* = 121)	
Emergency hospitalization, *n* (%)	57 (59.38)	110 (90.91)	0.001
Acute heart failure, *n* (%)	11 (11.46)	56 (46.28)	0.001
Zero time, *n* (%)	85 (88.54)	65 (53.72)	0.001
One time, *n* (%)	7 (7.29)	27 (22.31)	0.002
Two times, *n* (%)	2 (2.08)	20 (16.53)	0.001
Three times, *n* (%)	2 (2.08)	7 (5.79)	0.304
Four times, *n* (%)	0 (0.00)	3 (2.47)	0.257
Arrhythmia, *n* (%)	3 (3.13%)	4 (3.31%)	0.991
New onset acute coronary syndrome, *n* (%)	33 (34.38%)	38 (31.40%)	0.471
Stroke, *n* (%)	10 (10.42%)	12 (9.92%)	0.823

At the end of the follow up, 95 patients died (43.78%); of whom 47 (21.66%) patients died because of CVD. In the number of VI ≥ 2 group, 59 (48.76%) patients died, including 32 (26.45%) cases because of cardiovascular events. In the number of VI < 2 group, 36 (37.50%) patients died, containing 15 (15.63%) CVD cases.

### Risk factors of cardiovascular mortality, all-cause mortality and new onset ACS

To explore the independent risk factors of cardiovascular and all-cause mortality for the study patients, COX regression models were analyzed. The results showed that any single valve insufficiency was not related to cardiovascular or all-cause mortality. But age, number of VI ≥ 2 and ALB were all the independent risk factors of cardiovascular and all-cause mortality (Tables 3 and 4). Compared with number of VI < 2 group, number of VI ≥ 2 had higher cardiovascular mortality (HR 2.035, 95% CI 1.083–3.821, *P* = 0.027) and all-cause mortality (HR 1.711, 95% CI 1.102–2.656, *P* = 0.017).

**Table 3:** Univariate and multivariate cox proportional hazards analysis for cardiovascular mortality

	Univariate cox regression			Multivariate cox regression		
	HR	95% CI	*P*	HR	95% CI	*p* trend
Age, years	1.042	1.018–1.067	0.001	1.033	1.007–1.061	0.013
Sex, man	0.952	0.522–1.734	0.872	–	–	–
History of DM	1.979	1.074–3.646	0.029	1.380	0.720–2.644	0.332
History of CVD	2.462	1.636–3.705	0.000	1.667	0.897–3.097	0.106
History of hypertension	0.756	0.307–1.865	0.544	–	–	–
ACEI/ARB use, %	0.578	0.319–1.049	0.071	–	–	–
Mitral insufficiency	1.536	0.804–2.935	0.194	–	–	–
Tricuspid insufficiency	1.331	0.742–2.389	0.338	–	–	–
Aortic insufficiency	1.375	0.758–2.495	0.295	–	–	–
Pulmonary valve insufficiency	1.213	0.581–2.530	0.607	–	–	–
Number of VI ≥ 2	2.020	1.088–3.751	0.026	2.035	1.083–3.821	0.027
ALB (g/L)	0.920	0.874–0.967	0.001	0.935	0.881–0.992	0.027
Hemoglobin (Hb, g/L)	1.002	0.989–1.016	0.762	–	–	–
Ca (mmol/L)	0.909	0.301–2.747	0.866	–	–	–
P (mmol/L)	1.198	0.706–2.034	0.503	–	–	–
PTH (pg/mL)	0.999	0.998–1.000	0.108	–	–	–
Uric acid (mmol/L)	0.999	0.997–1.001	0.300	–	–	–

*DM* diabetes mellitus, *CVD* cardiovascular disease, *ACEI* angiotensin converting enzyme inhibitors, *ARB* angiotensin receptor blocker, *VI* valvular insufficiency, *ALB* serum albumin, *Ca* calcium, *P* phosphorus, *PTH* parathyroid hormone

**Table 4:** Univariate and multivariate cox proportional hazards analysis for all-cause mortality

	Univariate cox regression			Multivariate cox regression		
	HR	95% CI	*P*	HR	95% CI	*p* trend
Age, years	1.049	1.031–1.067	0.000	1.042	1.022–1.062	0.001
Sex, man	0.847	0.554–1.296	0.444	–	–	–
History of DM	1.899	1.245–2.897	0.003	1.279	0.800–2.044	0.304
History of CVD	2.570	1.428–4.625	0.002	1.558	0.996–2.438	0.052
History of hypertension	0.942	0.228–3.897	0.934	–	–	–
ACEI/ARB use, %	0.704	0.459–1.080	0.108	–	–	–
Mitral insufficiency	1.395	0.898–2.167	0.139	–	–	–
Tricuspid insufficiency	1.482	0.981–2.239	0.061	–	–	–
Aortic insufficiency	1.223	0.804–1.860	0.347	–	–	–
Pulmonary valve insufficiency	0.958	0.550–1.668	0.880	–	–	–
Number of VI ≥ 2	1.630	1.073–2.478	0.022	1.711	1.102–2.656	0.017
ALB (g/L)	0.926	0.893–0.960	0.000	0.949	0.908–0.991	0.019
Hemoglobin (Hb, g/L)	0.995	0.986–1.004	0.287	–	–	–
Ca (mmol/L)	0.779	0.364–1.669	0.521	–	–	–
P (mmol/L)	0.979	0.656–1.461	0.916	–	–	–
PTH (pg/mL)	0.999	0.998–1.000	0.012	1.000	0.999–1.001	0.459
Uric acid (mmol/L)	0.999	0.997–1.000	0.059	–	–	–

*DM* diabetes mellitus, *CVD* cardiovascular disease, *ACEI* angiotensin converting enzyme inhibitors, *ARB* angiotensin receptor blocker, *VI* valvular insufficiency, *ALB* serum albumin, *Ca* calcium, *P* phosphorus, *PTH* parathyroid hormone

To explore the relationship of VI and new onset ACS, COX regression models were analyzed. Unfortunately, none of VI or number of VI ≥ 2 was found to be correlated with new onset ACS. But we found that age (HR 1.023, 95% CI 1.004–1.043, *P* = 0.019), DM history (HR 1.686, 95% CI 1.040–2.734, *P* = 0.034) and CVD history (HR 2.271, 95% CI 1.395–3.697, *P* = 0.001) were independently associated with new onset ACS, as shown in Table 5.

**Table 5:** Univariate and multivariate cox proportional hazards analysis for new onset ACS

	Univariate cox regression			Multivariate cox regression		
	HR	95% CI	*P*	HR	95% CI	*p* trend
Age, years	1.036	1.018–1.055	0.000	1.023	1.004–1.043	0.019
Sex, man	0.843	0.523–1.360	0.485	–	–	–
History of DM	2.125	1.315–3.433	0.002	1.686	1.040–2.734	0.034
History of CVD	3.061	1.930–4.855	0.000	2.271	1.395–3.697	0.001
History of hypertension	0.569	0.229–1.414	0.225	–	–	–
ACEI/ARB use, %	0.838	0.508–1.383	0.489	–	–	–
Mitral insufficiency	0.917	0.573–1.467	0.717	–	–	–
Tricuspid insufficiency	1.127	0.710–1.789	0.612	–	–	–
Aortic insufficiency	1.035	0.642–1.669	0.888	–	–	–
Pulmonary valve insufficiency	0.575	0.275–1.201	0.141	–	–	–
Number of VI ≥ 2	1.012	0.639–1.604	0.959	–	–	–
ALB (g/L)	0.968	0.929–1.010	0.131	–	–	–
Hemoglobin (Hb, g/L)	1.008	0.997–1.018	0.155	–	–	–
Ca (mmol/L)	1.465	0.595–3.611	0.407	–	–	–
P (mmol/L)	0.793	0.498–1.264	0.329	–	–	–
PTH (pg/mL)	1.000	0.999–1.000	0.272	–	–	–
Uric acid (mmol/L)	1.000	0.998–1.001	0.800	–	–	–

*DM* diabetes mellitus, *CVD* cardiovascular disease, *ACEI* angiotensin converting enzyme inhibitors, *ARB* angiotensin receptor blocker, *VI* valvular insufficiency, *ALB* serum albumin, *Ca* calcium, *P* phosphorus, *PTH* parathyroid hormone

To further find the influencing factors of VI ≥ 2, multivariate Logistic regression was used. The analysis results showed that after adjusting for multiple factors, only hemoglobin was related to VI ≥ 2 (OR 0.982, 95% CI 0.967–0.996, *P* = 0.015), as shown in Table 6.

**Table 6:** Multivariate logistic regression analysis for VI ≥ 2

	Multivariate logistic regression		
	OR	95% CI	*p* trend
Age, years	0.997	0.975–1.02	0.793
Sex, man	0.766	0.415–1.415	0.395
History of DM	0.588	0.300–1.150	0.121
History of CVD	1.776	0.891–3.541	0.103
History of hypertension	0.729	0.145–3.660	0.701
ACEI/ARB use, %	0.708	0.346–1.446	0.343
ALB (g/L)	0.982	0.924–1.043	0.547
Hemoglobin (Hb, g/L)	0.982	0.967–0.996	0.015
Ca (mmol/L)	0.542	0.147–2.000	0.358
P (mmol/L)	0.855	0.469–1.558	0.609
PTH (pg/mL)	1.000	0.999–1.001	0.724
Uric acid (mmol/L)	0.999	0.997–1.001	0.452

*DM* diabetes mellitus, *CVD* cardiovascular disease, *ACEI* angiotensin converting enzyme inhibitors, *ARB* angiotensin receptor blocker, *VI* valvular insufficiency, *ALB* serum albumin, *Ca* calcium, *P* phosphorus, *PTH* parathyroid hormone

### Kaplan–Meier analyses

**Fig. 1:**
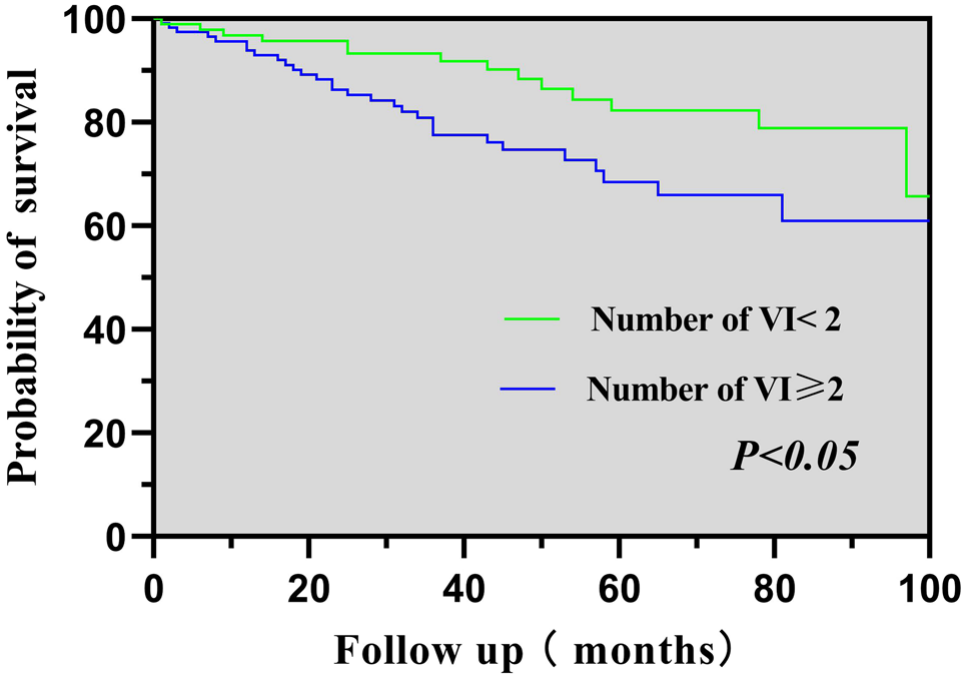
K-M analysis for cardiovascular

**Fig. 2:**
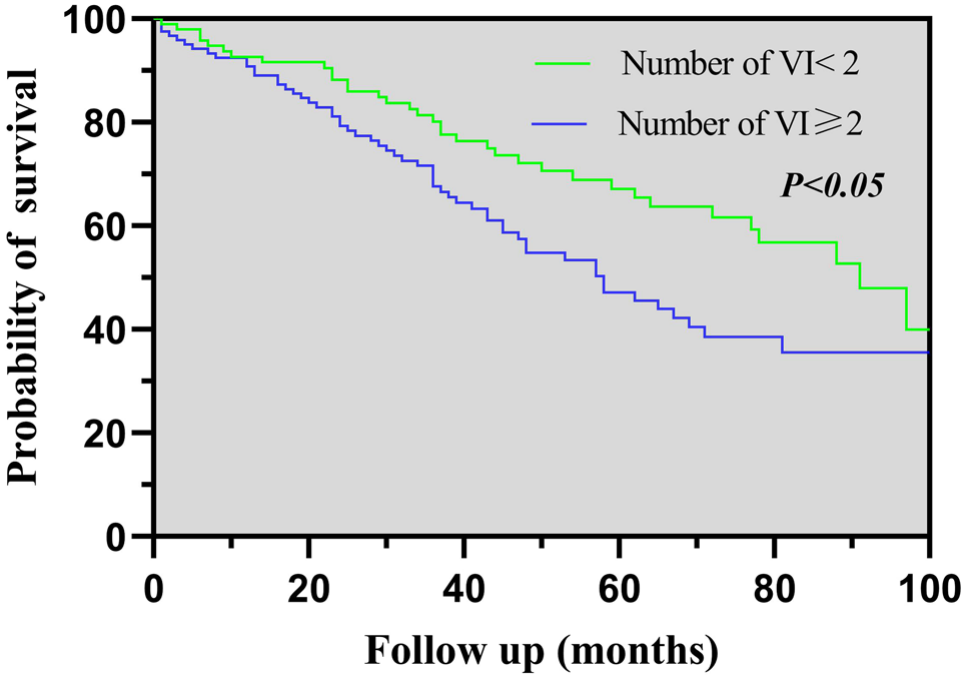
K-M analysis for all-cause mortality

To compare the difference in survival between the two groups, Kaplan–Meier analyses were performed. Kaplan–Meier analysis of cardiovascular mortality results showed that number of VI ≥ 2 group had a significantly lower probability of survival than number of VI < 2 group (*χ*^2^ = 6.839, *P* = 0.009), as shown in Fig. 1. Figure 2 showed similar results in all-cause mortality (χ^2^ = 5.604, *P* = 0.018).

### Nomogram models for 5-year cardiovascular and all-cause mortality

**Fig. 3:**
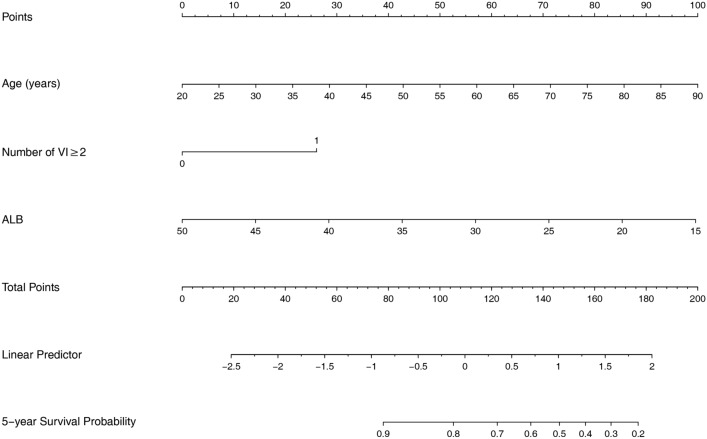
Nomogram model for 5-year cardiovascular mortality

**Fig. 4:**
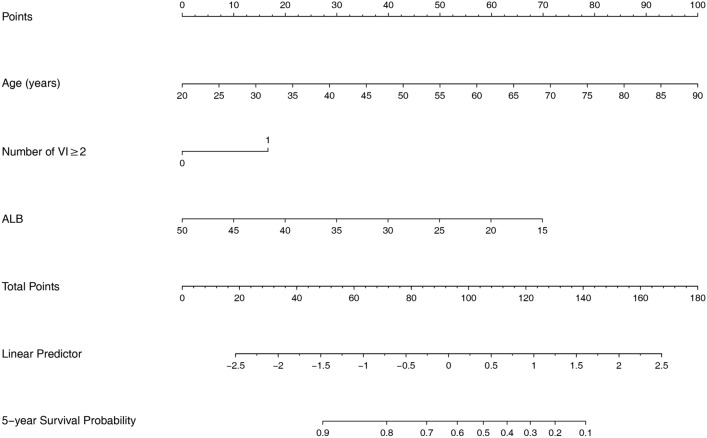
Nomogram model for 5-year all-cause mortality

**Fig. 5:**
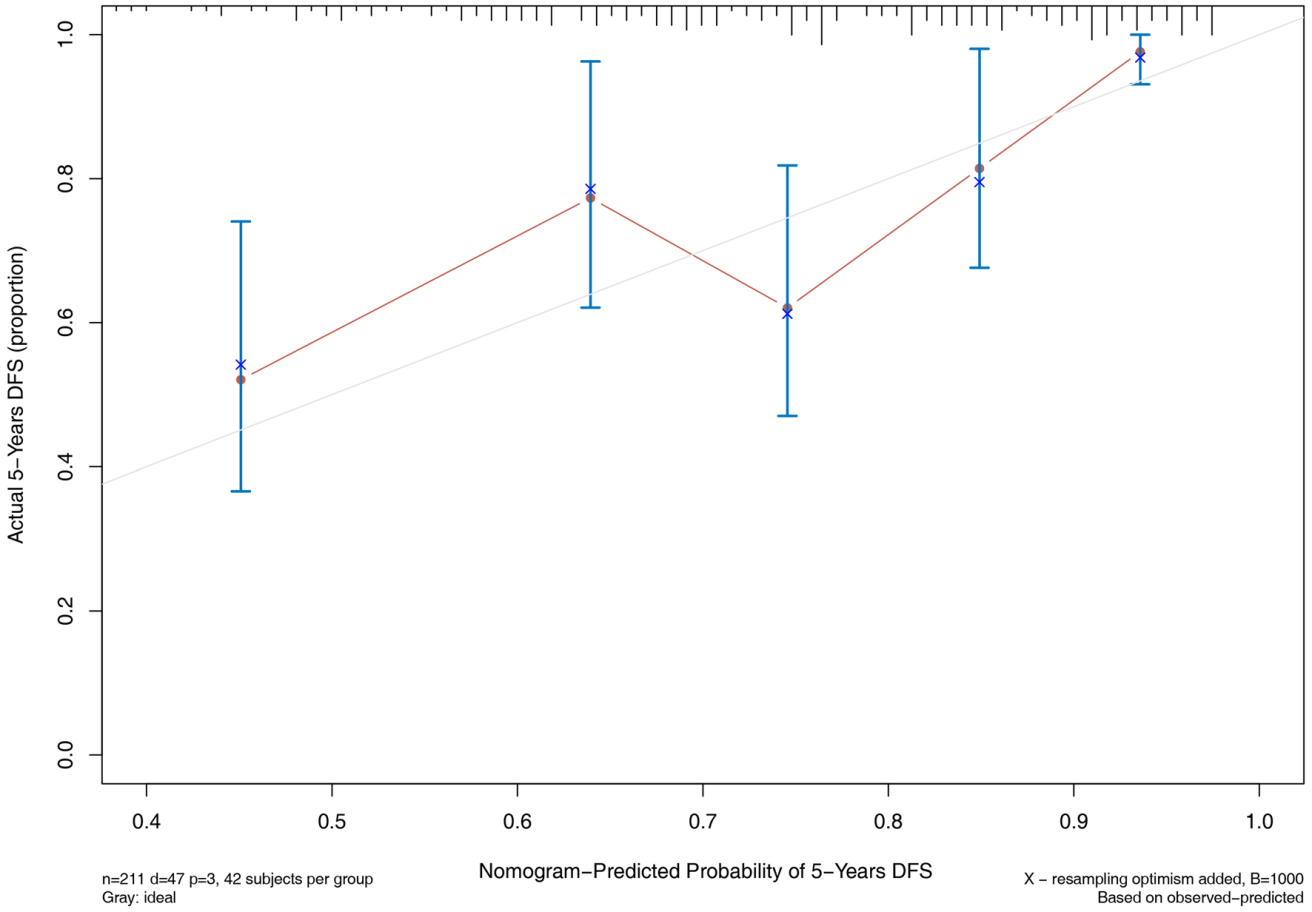
Calibration curve for cardiovascular mortality

**Fig. 6:**
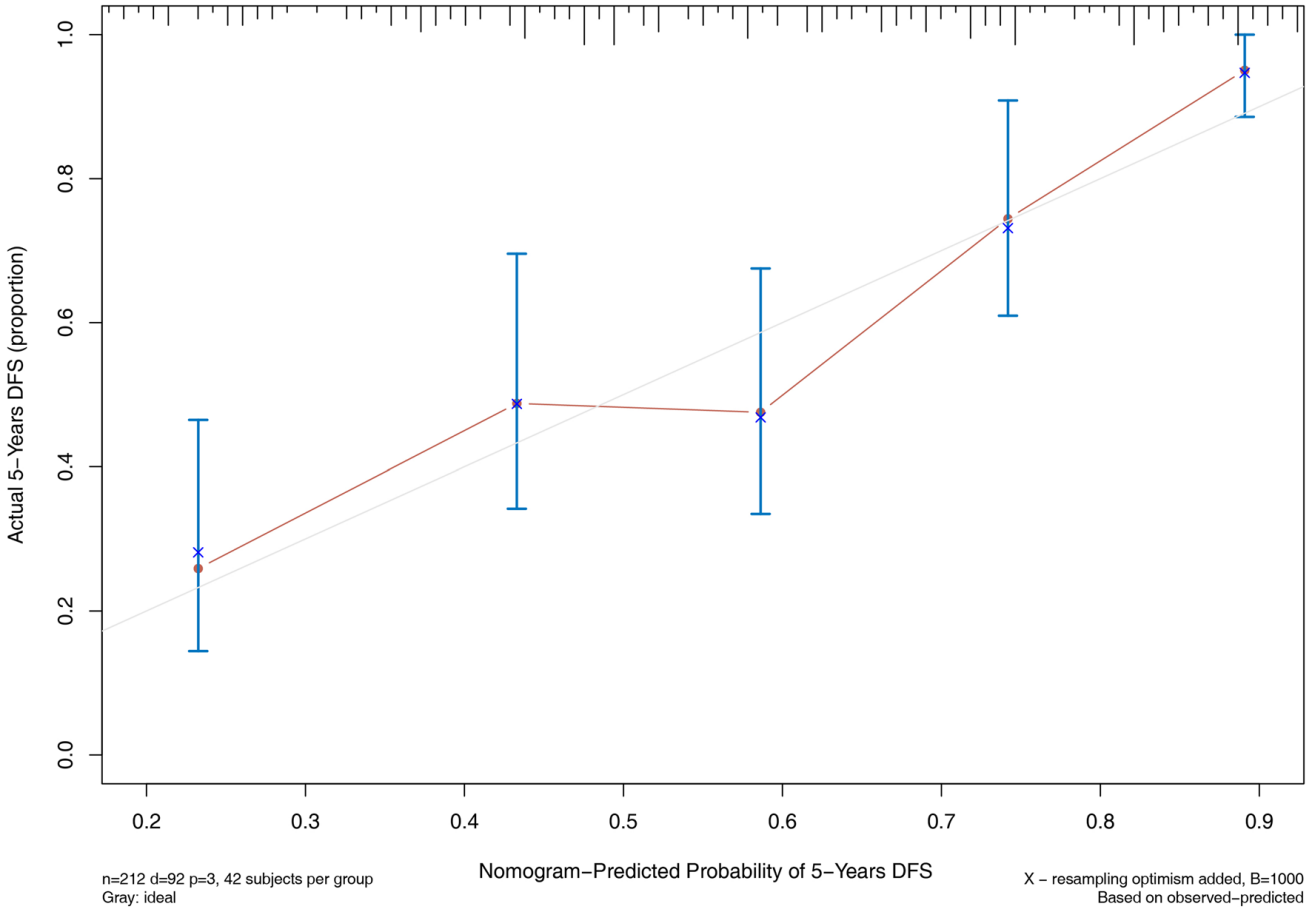
Calibration curve for all-cause mortality

According to the results of multivariate COX regression analysis, we built the nomogram models to predict 5-year cardiovascular and all-cause mortality. Three variables from the independent risk factors were included: age, number of VI ≥ 2 and ALB. Based on the individual scores calculated using the nomogram, a total score was determined (Figs. 3, 4). The C-index of 5-year cardiovascular mortality model was 0.698 and the C-index for 5-year all-cause mortality model was 0.703. The calibration curves of two nomogram models are shown in Figs. 5, 6.

## Discussion

Our study showed that the morbidity of VI was common in maintenance HD patients, accounting for 81.57%. And 64.98% patients had mitral insufficiency, which was the most common. Mitral insufficiency was also the first valve disease in the general population [15]. A retrospective analysis of 1326 dialysis patients found 42.8% cases had mitral regurgitation, in which 30.4% mild, 9.7% moderate, 2.6% severe [5]. In our study, the morbidity of tricuspid insufficiency was 53.46%, which was second. Tricuspid regurgitation was also a common valvular disease with high morbidity and mortality in general population [16, 17]. Unfortunately, no large-scale studies have reported the incidence of tricuspid regurgitation in dialysis patients. In general, VI can be classified as primary or secondary. Primary causes are structural or degenerative abnormalities at any part of the valve apparatus, containing leaflets, chordae tendineae, papillary muscles or annulus. Secondary causes are associated with heart geometrical alterations, usually from cardiac dysfunction without organic valvular diseases. Unfortunately, our study did not further explore the causes of VI in the research subjects.

In our study, 49.47% death cases were because CVD. Over the past decade, with the development of medical technology and medical insurance, the quality of life and survival rate of maintenance HD patients have been significantly improved. Ultra-pure dialysis water, dialyzers with high biocompatibility and clearance rate, individualized dialysis programs, focusing on nutrition and complication control, all make important contributions. Despite this, CVD remains the most common cause of mortality in maintenance HD patients. Evidence showed that the risk of premature death due to CVD in maintenance HD patients was 20 times higher than in the general population [18].

Samad et al.’s research showed that 5-year survival in patients decreased as mitral regurgitation severity increasing [5]. Green et al. reported mitral regurgitation was the only parameters predictive of sudden cardiac death [19]. Based on the data analysis of 533 patients admitted for first systolic heart failure, Hsiao SH et al. also discovered that systolic pulmonary regurgitation was associated with cardiovascular death [20]. Nevertheless, we found that insufficiency of any one valve was not the independent risk factor for cardiovascular mortality or all-cause mortality, but number of VI ≥ 2 was correlated to both. It means in our future work, we need to pay more attention to the number of valvular lesions in dialysis patients. In addition, we also found that age and albumin were independent risk factors for cardiovascular and all-cause death in maintenance HD patients. A meta-analysis for patients undergoing hemodialysis also found that age and lower albumin were the risk factors of cardiac death [21]. Based on our results, we build the nomogram models to predict 5-year cardiovascular and all-cause mortality.

Some researches revealed DM and previous CVD were also correlated to cardiovascular and all-cause mortality [21, 22]. But in our results, neither history of DM nor CVD was associated with cardiovascular or all-cause mortality. Banshodani et al. analyzed 260 dialysis patients and also discovered that DM was not an independent risk factor for cardiovascular or all-cause mortality [23]. This difference may be related to the sample size.

In addition, no research has reported the relationship between the number of VI and acute heart failure. For the first time, we found that maintenance HD patients with number of VI ≥ 2 were more likely to be emergency hospitalized for acute heart failure. On the contrary, the number of VI was not associated with emergency hospitalized for arrhythmia, ACS or stroke. Mitral regurgitation can leads to heart failure by inducing volume overload of the heart [24]. Systolic pulmonary regurgitation was also considered to correlate with heart failure rehospitalization [20]. But a study of 639 hospitalized patients with acute heart failure found that moderate or severe tricuspid regurgitation was not associated with readmission for heart failure [25].

It is well known that ischemic mitral regurgitation is frequently associated with AMI [26]. On the contrary, whether patients with VI are more likely to develop ACS has not been reported. In our study, we found neither any VI nor number of VI ≥ 2 was the independent risk of new onset ACS in maintenance HD patients.

Anemia is a common complication in maintenance HD patients. Our study found that hemoglobin was associated with more than two valvular lesions, but the specific mechanism needed further study. Tigen et al.’ study proved anemia (hemoglobin levels less than 12.5 mg/dL) was an independent predictor of moderate or severe functional mitral regurgitation in non-ischemic dilated cardiomyopathy patients with normal renal function [27].

There are several limitations in our study: (1) This is a retrospective study with a single-center. The sample size is small, which may lead to selective bias. (2) Some covariates that might affect cardiovascular and all-cause mortality in maintenance HD patients have not been included, such as spKt/V homocysteine dialysis vintage. (3) Severity of valvular insufficiency has not been subdivided, which may have more value for cardiovascular and all-cause mortality.

In conclusion, the prevalence of VI in maintenance HD patients is very high. The number of VI ≥ 2 is associated with emergency hospitalized for acute heart failure, cardiovascular, and all-cause mortality in maintenance HD patients. In addition, combining age, number of VI ≥ 2 and albumin can predict 5-year cardiovascular and all-cause mortality in maintenance HD patients.

## Data Availability

All data that support the findings of this study are available from the corresponding author upon reasonable request.
